# Impact of perceived side-effects of psychotropic treatments on quality of life in patients with severe mental illness

**DOI:** 10.1080/19585969.2025.2463443

**Published:** 2025-02-11

**Authors:** Théo Korchia, Mélanie Faugère, Vincent Achour, Eloïse Maakaron, Christelle Andrieu-Haller, Guillaume Fond, Christophe Lançon

**Affiliations:** ^a^ Department of University Psychiatry, Sainte Marguerite University Hospital, Assistance Publique des Hôpitaux de Marseille, Marseille, France; ^b^ Assistance Publique des Hôpitaux de Marseille, Aix-Marseille University, UR3279: Health Service Research and Quality of Life Center – CEReSS, Marseille, France

**Keywords:** Psychotropic treatments, side effects, quality of life, severe mental illness

## Abstract

**Background:**

Psychotropic medications are critical in managing severe mental illnesses (SMI) such as schizophrenia, major depressive disorder (MDD) and bipolar disorder. However, these treatments often lead to adverse side effects that can impair patients’ quality of life (QoL) and affect treatment adherence.

**Objective:**

This study aims to investigate the specific side effects of psychotropic treatments that contribute to a decline in QoL among patients with SMI, independently of treatment adherence.

**Methods:**

We conducted a cross-sectional study with 1248 patients diagnosed with SMI, recruited from a university psychiatric unit in Marseille, France. QoL was assessed using the Schizophrenia Quality of Life Scale (SQoL-18), and side effects were measured using the UKU Side Effect Rating Scale. Treatment adherence was evaluated using the Medication Adherence Rating Scale (MARS). Statistical analyses included Pearson correlations and multiple linear regression models to identify predictors of QoL.

**Results:**

The study found that side effects, as identified by the UKU scores, could significantly predict a reduction in QoL across multiple domains, including multiple dimensions of QoL and the overall QoL index, independent of treatment adherence. Patients on antipsychotics and benzodiazepines reported higher levels of adverse side effects, which correlated with lower QoL scores. An increase in the number of psychotropic treatment classes was also associated with a significant decline in QoL (*p* < 0.001).

**Conclusion:**

Managing psychic side effects and minimising polypharmacy are critical to improving QoL in patients with SMI. Clinicians should consider these factors when developing personalised treatment strategies to enhance patient outcomes.

## Introduction

The impact of psychiatric disorders on patients’ quality of life (QoL) is a significant concern due to their chronic nature. Conditions such as schizophrenia, major depressive disorder (MDD) and bipolar disorder not only affect mental health but also leads to physical health problems (Schuch and Vancampfort 2021), social difficulties (Høegh et al. 2022), overall well-being and emotional distresses (Kraiss et al. 2020). These disorders impose a substantial socioeconomic burden (Kotzeva et al. 2023). While pharmacological treatments are essential for managing these conditions, they can cause a range of side effects, further complicating treatment and impacting QoL (Pillinger et al. 2023).

Antipsychotic medications, essential in the management of schizophrenia, bipolar disorder and MDD, are particularly known for their side effects (Huhn et al. 2019). These medications, although effective in reducing psychotic symptoms, are associated with a variety of adverse effects, including neurological (e.g. extrapyramidal symptoms), metabolic (e.g. weight gain, metabolic syndrome), sexual disorders (Korchia et al. 2023) and autonomic (e.g. orthostatic hypotension) disturbances. These side effects can significantly impair QoL and reduce treatment adherence (Leucht et al. 2023). Leucht et al. found substantial variability in the side effect profiles of different antipsychotic drugs in a recent article (Leucht et al. 2013). Correll et al. highlighted the metabolic risks associated with atypical antipsychotics (Correll et al. 2006). Additionally, psychiatric comorbidities such as generalised anxiety disorder (Beard and Björgvinsson 2014), panic disorder (Ophuis et al. 2018) and post-traumatic stress disorder (Schein et al. 2021) are common among patients with severe mental illnesses, exacerbating the burden of side effects and further impacting QoL. Buckley et al. discussed the intricate relationship between psychiatric comorbidities and schizophrenia (Buckley et al. 2009), while Hirschfeld provided insights into the challenges of managing comorbid depression and anxiety in primary care settings (Hirschfeld 2001).

The UKU Side Effect Rating Scale is a widely used tool for assessing the side effects of psychotropic medications (Lingjaerde et al. 1987). This scale categorises side effects into psychic, neurological, autonomic and other categories, allowing for a comprehensive evaluation of the impact of these medications on patients. Research has consistently shown that side effects significantly affect patients’ quality of life. For example, Lindenmayer et al. found 20 years ago that side effects were a major determinant of patients’ quality of life (Lindenmayer et al. 2004) and Awad and Voruganti highlighted the impact of side effects on caregivers and their association with patient outcomes (Awad and Voruganti 2008).

Given the critical impact of side effects on quality of life, this study aims to identify specific side effects of psychotropic treatments associated with a deterioration in QoL among patients with severe mental illness (SMI), independently of treatment adherence, as measured by the Medication Adherence Rating Scale (MARS) (Fond et al. 2017). We will also investigate which classes of psychotropic treatments are most associated with side effects and a decline in QoL. Additionally, we will examine whether an increase in the number of psychotropic treatment classes, particularly benzodiazepines, is linked to a decrease in QoL due to their prevalent use and potential for adverse effects (de la Iglesia-Larrad et al. 2020).

This study investigates the complex interplay between psychotropic medication side effects and quality of life in a large cohort of patients with SMI. By elucidating the relationship between psychotropic medication side effects and QoL, we aim to optimise therapeutic strategies to enhance patient well-being and treatment adherence.

## Materials and methods

### Study

This study was conducted in a day hospital at in a university psychiatry unit, located in Marseille, France. Patients were recruited over a 2-year period from January 2020 to December 2021.

### Participants

The participants were patients diagnosed with SMI, including schizophrenia, bipolar disorder and major depressive disorder (MDD). Inclusion criteria required participants to have a diagnosis according to DSM-5 criteria, be aged between 18 and 65 years, and have a regular follow-up at the day hospital for at least 6 months. Exclusion criteria included major neurocognitive disorders, severe unstable organic comorbidities and inability to understand or complete the study questionnaires. Patients aged over 65 were excluded due to the higher likelihood of comorbidities and cognitive decline, which could possibly affect the accuracy of the self-reported data.

### Sampling

We included all the patients coming for regular evaluations at the day hospital over a 2-year period. Patients came first for clinical exams and medical advice, then we collected data during routine clinical evaluations and structured interviews.

### Procedure

All participants were evaluated during regular consultations at the day hospital. Demographic and clinical data were collected at the time of study inclusion, and the SQoL-18, UKU, MARS and others clinical scales were administered by trained healthcare professionals. Data collection involved semi-structured interviews and review of patients’ medical records. Each patient completed the questionnaires under the supervision of a clinician to ensure understanding and accuracy of responses. Data quality was ensured through periodic quality assurance checks which were performed by a second researcher who reviewed 20% of the data entries randomly selected for consistency with source documentation. These checks were intended to ensure data accuracy and reduce errors during the entry process.

### Measurement tools

#### SQoL-18

Quality of life was assessed using the SQoL-18 scale, a self-administered and multidimensional questionnaire assessing quality of life and initially developed for individuals with schizophrenia (Boyer et al. 2010). The questionnaire include 18 items describing 8 dimensions (Psychological Well-being, Self-Esteem, Family Relationships, Relationships with Friends, Resilience, Physical Well-being, Autonomy and Sentimental Life). It also includes a total score (Index). The eight dimensions and the index score range from 0 to 100, higher scores indicate better quality of life. The questionnaire had also been validated in bipolar and depressive disorders, allowing trans-diagnostical analysis (Boyer et al. 2022).

#### UKU

Side effects of psychotropic treatments were evaluated using the UKU Side Effect Rating Scale (Lingjaerde et al. 1987), one of the most known instruments to assess side effects. Using a semi-structured interview, this scale measures 48 individuals side effects grouped in 4 domains: psychic (I), neurological (II), autonomic (III) and others (IV) side effects. Each item is scored from 0 to 3 and higher scores indicate higher degrees of side effects. The UKU is an inclusive instrument with high reported reliability and validity. The side effects were based on patients’ perceptions, which could potentially be influenced by underlying comorbidities or primary psychiatric symptoms.

UKU can be altered by different types of treatment and it seems difficult to make individual links, as several treatments affect several areas of the UKU. However, it seems logical to assume that psychic side effects (UKU I) are due to antipsychotics side effects: difficulty concentrating, asthenia, fatigue, sedation, memory difficulties, tension, sleep changes… Neurological side effects (UKU II) seems logically attributed to antipsychotics too (dystonia, akathisia, paraesthesia, etc.). Autonomic side effects (UKU III) could be attributed to all the psychotropic medications (change in visual accommodation, nausea, diarrhoea, palpitations…). And others side effects (UKU IV) could also be attributed to all the psychotropic medications (erythema, itchiness, weight changes, amenorrhea…).

#### MARS

Treatment adherence was measured using the Medication Adherence Rating Scale (MARS), a self-reported scale that assesses patients’ adherence towards their medication. The MARS is a questionnaire of 10 items, each coded as a ‘yes or no question’. Higher total scores indicate better adherence to medication.

#### CGI-S

Illness severity was measured using the Clinical Global Impressions - severity scale (CGI-S; Guy 1976), ranked from 0 to 7: higher scores indicate higher severity of the disease.

#### CDSS

The depressive symptoms were assessed by the Calgary Depression Scale for Schizophrenia (CDSS; Bernard et al. 1998), a nine-item structured interview scale designed to assess depression in schizophrenics, validated in mood disorders (Micoulaud-Franchi et al. 2018). Each item is ranked from 0 to 3, and higher scores indicate higher depressive symptoms severity.

#### STAI-YA

Anxiety levels were assessed by the State-Trait Anxiety Inventory (STAI-YA) (Knowles and Olatunji 2020), made of 20 questions ranked from 1 to 4. Higher scores indicate higher anxiety levels.

### Statistical analysis

Statistical analyses were performed using SPSS software (version 20.0). Firstly, we described the population with means, standard deviations, frequencies and percentages of the demographic and clinical variables, including the mean scores for the SQoL-18, UKU, MARS and others clinical scales. Then, we used Pearson correlations to explore relationships between side effects (UKU) and quality of life (SQoL-18). Finally, multiple linear regression analyses were conducted to identify if UKU domains were predictors of quality of life dimensions, controlling for demographic (age, sex, education level) and clinical variables (diagnostic, MARS and CGI-S scores). Additional multiple linear regression analyses were also conducted, controlling for the same factors, and we added antidepressants (SSRIs), antipsychotics (typical and atypical) and benzodiazepines, to best account for comorbidities, notably anxiety and depression.

### Ethical considerations

The study was approved by the ethics committee of the Marseille day hospital. All procedures were conducted in accordance with the ethical principles of the Declaration of Helsinki and the Jardé law. Patient data were anonymised to ensure confidentiality and protection of personal information. All participants provided written informed consent to participate in the study, in accordance with the Declaration of Helsinki, the Jardé law, and the ethical guidelines of the Marseille day hospital’s ethics committee.

## Results

### Demographic and clinical characteristics

A total of 1248 participants with SMI were included in the study. The mean age of the participants was 39.66 years (SD = 14.33), with 57.9% men (*n* = 723) and 42.1% women (*n* = 525). Education level averaged 12.51 years (SD = 3.08), with 65.1% (*n* = 555) of participants having more than 12 years of education. The majority of participants were single (63.8%, *n* = 783) and 19.8% (*n* = 243) were part of the working force (Table 1).

**Table 1.** Description of the sample.

Sociodemographic characteristics of the sample (*N* = 1248)	Mean (SD) or N (%)
Gender	
Men	723 (57.9%)
Women	525 (42.1%)
Age (years)	39.66 (14.33)
Education level	12.51 (3.08)
Education level >12 years	555 (65.1%)
Single	783 (63.8%)
Working force status	243 (19.8%)
Diagnostic	
Schizophrenia	554 (44.4%)
Major depressive disorder	533 (42.7%)
Bipolar disorder	161 (12.9%)
Medical psychiatric history	
Age at illness onset	24.43 (11.52)
Disease duration	12.39 (10.43)
History of suicide attempt	154 (25.3%)
Psychiatric comorbidities	
General anxiety disorder	411 (33.4%)
Panic disorder	257 (20.9%)
Agoraphobia	207 (16.8%)
Social phobia	212 (17.2%)
Post-traumatic stress disorder	70 (5.7%)
Physical health	
Smokers	639 (54.1%)
Tobacco (PA)	14.44 (15.29)
Fagerström score	4.71 (2.97)
Current cannabis	222 (18.8%)
Current alcohol abuse	205 (17.5%)
Metabolism	
Hypertension	465 (37.5%)
Hyperglycaemia	153 (12.6%)
Hypertriglyceridaemia	271 (22.5%)
Low HDL	280 (23.6%)
Abdominal perimeter	743 (62.1%)
Metabolic syndrome	269 (22.5%)
Body mass index	25.64 (5.59)
Obesity	240 (19.4%)
Treatments	
Psychotropic	
Antipsychotics	648 (51.9%)
Atypical	614 (49.2%)
Atypical, long acting	84 (6.7%)
Typical	109 (8.7%)
Chlorpromazine equivalent (mg/day)	407.84 (639.91)
Antidepressants	606 (48.6%)
SSRI	334 (26.8%)
SNRI	204 (16.3%)
Alpha 2	66 (5.3%)
Mood stabilisers	119 (9.5%)
Antiepileptic	58 (4.6%)
Benzodiazepines	383 (30.7%)
Hypnotics	79 (13.7%)
Clinical scales	
CDSS score	5.77 (5.56)
CDSS cut-off	270 (42.4%)
MARS	6.17 (2.31)
STAI-YA	49.57 (13.88)
CGI-S	4.19 (1.19)
UKU I	5.60 (4.89)
UKU II	1.18 (1.55)
UKU III	2.82 (2.79)
UKU IV	3.77 (4.16)
SQoL-18	
Psychological well-being	50.66 (28.73)
Self esteem	44.50 (30.72)
Relations with family	64.40 (29.30)
Relations with friends	48.65 (30.30)
Resilience	53.73 (28.04)
Physical well-being	40.49 (28.88)
Autonomy	57.36 (28.73)
Sentimental life	35.93 (32.10)
SQoL-18 index	48.98 (19.61)

### Diagnostic and psychiatric history

Among participants, schizophrenia was the main represented diagnostic (44.4%, *n* = 554), followed by MDD (42.7%, *n* = 533) and bipolar disorder (12.9%, *n* = 161). The mean age at illness onset was 24.43 years (SD = 11.52), with an average disease duration of 12.39 years (SD = 10.43). A history of suicide attempts was reported by 25.3% (*n* = 154) of participants (Table 1).

### Psychiatric comorbidities

The main psychiatric comorbidities were generalised anxiety disorder (33.4%, *n* = 411), panic disorder (20.9%, *n* = 257), agoraphobia (16.8%, *n* = 207), social phobia (17.2%, *n* = 212) and PTSD (5.7%, *n* = 70) (Table 1).

### Physical health and substance use

Regarding physical health, 54.1% (*n* = 639) of participants were smokers, with a mean tobacco use of 14.44 pack-years (SD = 15.29). Current cannabis use was reported by 18.8% (*n* = 222) of participants and 17.5% (*n* = 205) reported current alcohol abuse. Metabolic issues were common, with 37.5% (*n* = 465) having hypertension, 12.6% (*n* = 153) having hyperglycaemia, 22.5% (*n* = 271) hypertriglyceridaemia, 23.6% (*n* = 280) low HDL cholesterol and 19.4% (*n* = 240) obesity. Metabolic syndrome was present in 22.5% (*n* = 269) of participants. Mean BMI was 25.64 (SD = 5.59) (Table 1).

### Psychotropic treatments

A significant part of participants (51.9%, *n* = 648) were on antipsychotics, 49.2% (*n* = 614) took atypical antipsychotics and 6.7% (*n* = 84) long-acting atypical antipsychotics. Antidepressants were used by 48.6% (*n* = 606) of participants, with selective serotonin reuptake inhibitors (SSRIs) being the most represented (26.8%, *n* = 334) before serotonin and norepinephrine reuptake inhibitors (SNRIs) (16.3%, *n* = 204). Other psychotropic medications included mood stabilisers (9.5%, *n* = 119), anti-epileptics (4.6%, *n* = 58), benzodiazepines (30.7%, *n* = 383) and hypnotics (13.7%, *n* = 79) (Table 1).

### Clinical scales

The mean score on the CDSS was 5.77 (SD = 5.56), with 42.4% (*n* = 270) of participants exceeding the clinical cut-off for depression. The MARS had a mean score of 6.17 (SD = 2.31) and the STAI-YA showed a mean score of 49.57 (SD = 13.88). The CGI-S had a mean score of 4.19 (SD = 1.19) (Table 1).

### Side effects

Among our sample, the mean score of the UKU I (psychic side effects) was 5.60 (SD = 4.89), 1.18 (SD = 1.55) for the UKU II (neurological side effects), 2.82 (SD = 2.79) for the UKU III (autonomic side effects) and 3.77 (SD = 4.16) for the UKU IV (others side effects).

### Quality of life (SQoL-18)

Quality of life was assessed using the SQoL-18 scale, with the following mean scores: Psychological Well-being (PsW) 50.66 (SD = 28.73), Self-Esteem (SE) 44.50 (SD = 30.72), Relationships with Family (RFa) 64.40 (SD = 29.30), Relationships with Friends (RFr) 48.65 (SD = 30.30), Resilience (RE) 53.73 (SD = 28.04), Physical Well-being (PhW) 40.49 (SD = 28.88), Autonomy (AU) 57.36 (SD = 28.73) and Sentimental Life (SL) 35.93 (SD = 32.10). The overall SQoL-18 Index score was 48.98 (SD = 19.61) (Table 1).

### Correlations with quality of life

Correlation analyses revealed significant associations between quality of life scores and various factors. Psychic side effects (UKU I) were negatively correlated with the SQoL-18 Index (r = –0.311, *p* < 0.01), indicating that higher psychic side effects were associated with lower quality of life. Neurological side effects (UKU II) showed weaker but still notable negative correlations with the SQoL-18 Index (r = –0.094, *p* < 0.05). Autonomic side effects (UKU III) and other side effects (UKU IV) also negatively correlated with various quality of life domains, though these correlations were generally weaker (Table 2).

**Table 2.** Pearson’s correlations between UKU and SQoL-18 scores.

SQoL-18 (R)	Psychological well-being	Self esteem	Relations with family	Relations with friends	Resilience	Physical well-being	Autonomy	Sentimental life	SQoL-18 index
UKU I	**−0.304** **	**−0.294** **	−0.27	**−0.137** **	**−0.197** **	**0.305** **	**−0.225** **	**−0.172** **	**−0.311** **
UKU II	−0.077	−0.079	0.012	−0.085	−0.0.24	−0.079	**−0.102** *	0.013	**−0.094** *
UKU III	**−0.196** **	**−0.214** **	−0.025	−0..068	−0.063	**−0.240** **	**−0.143** **	−0.051	**−0.173** **
UKU IV	−0.062	**−0.093** *	−0.019	−0.074	−0.021	**−0.165** **	−0.086	−0.042	**−0.094** * ^a^

*p < 0.05.

**p < 0.01.

^a^ *p* < 0.05 results in bold

The detailed breakdown showed that psychic side effects had the most significant negative correlations across multiple domains of the SQoL-18, including Psychological Well-being (r = –0.304, *p* < 0.01), Self-Esteem (r = –0.294, *p* < 0.01) and Sentimental Life (r = –0.–172, *p* < 0.01). Autonomic side effects also negatively impacted Psychological Well-being (r = –0.196, *p* < 0.01) and Physical Well-being (r = –0.240, *p* < 0.01) (Table 2).

### Regression analyses

Multiple regression analyses identified significant predictors of quality of life. Age was a significant negative predictor of Relationships with Family (β = –0.272, *p* ≤ 0.001), Resilience (β = –0.165, *p* = 0.010) and Autonomy (β = –0.129, *p* = 0.036). Psychic side effects (UKU I) were consistently significant negative predictors across several quality of life domains, including Psychological Well-being (β = –0.260, *p* < 0.001), Self-Esteem (β = –0.136, *p* = 0.043), Resilience (β = –0.212, *p* = 0.003), Physical Well-Being (β = –0.175, *p* = 0.012), Autonomy (β = –0.185, *p* = 0.007) and the overall SQoL-18 Index (β = –0.240, *p* < 0.001). Others Side Effects (UKU IV) were a significant positive predictor of Psychological Well-Being (β = 0.125, *p* = 0.048). Treatment adherence (MARS) positively predicted all the quality of life dimensions: Psychological Well-Being (β = 0.115, *p* = 0.036), Self-Esteem (β = 0.219, *p* < 0.001), Relationships with Family (β = 0.143, *p* = 0.017), Relationships with Friends (β = 0.126, *p* = 0.039), Resilience (β = 0.174, *p* = 0.002), Physical Well-Being (β = 0.125, *p* = 0.027), Autonomy (β = 0.161, *p* = 0.004), Sentimental Life (β = 0.139, *p* = 0.025) and the overall SQoL-18 Index (β = 0.202, *p* < 0.001). Diagnostic category also emerged as a significant predictor, with those diagnosed with MDD or bipolar disorder showing different patterns compared to those with schizophrenia. Illness severity (measured by the CGI-S) was a negative predictor of all the SQoL-18 dimensions (and the overall Index), except the Relationships with Family (Table 3).

**Table 3.** multiple linear regressions analyses of relation between different side effects (UKU) and quality of life (SQoL-18).

	Psychological well-being		Self esteem		Relations with family		Relations with friends		Resilience	
SQoL-18 dimensions	Standardized bêta	*p*	Standardized bêta	*p*	Standardized bêta	*p*	Standardized bêta	*p*	Standardized bêta	*p*
Age	0.013	0.835	0.032	0.601	**−0.272**	**<0.001**	0.032	0.632	**−0.165**	**0.010**
Sex	−0.018	0.737	−0.037	0.507	−0.039	0.527	−0.033	0.591	0.079	0.173
Education level	−0.012	0.818	0.017	0.735	−0.045	0.432	−0.042	0.470	0.011	0.837
UKU I	**−0.260**	**<0.001**	**−0.136**	**0.043**	−0.049	0.512	−0.061	0.419	**−0.212**	**0.003**
UKU II	−0.016	0.776	−0.012	0.829	0.043	0.497	−0.030	0.638	−0.015	0.800
UKU III	−0.023	0.723	−0.012	0.852	−0.025	0.729	−0.032	0.660	0.025	0.716
UKU IV	**0.125**	**0.048**	0.034	0.596	0.060	0.401	−0.001	0.993	0.069	0.304
MARS	**0.115**	**0.036**	**0.219**	**<0.001**	**0.143**	**0.017**	**0.126**	**0.039**	**0.174**	**0.002**
Diagnostic*	**0.216**	**<0.001**	**0.260**	**<0.001**	−0.125	0.059	−0.013	0.841	0.057	0.361
CGI-S	**−0.304**	**<0.001**	**−0.272**	**<0.001**	−0.079	0.213	**−0.182**	**0.005**	**−0.182**	**0.003** ^a^

^a^ *p* < 0.05 results in **bold**

**Table:** 

	Physical well-being		Autonomy		Sentimental life		SQoL-18 index	
SQoL-18 dimensions	Standardized bêta	*p*	Standardized bêta	*p*	Standardized bêta	*p*	Standardized bêta	*p*
Age	−0.108	0.086	**−0.129**	**0.036**	0.036	0.596	−0.100	0.090
Sex	0.025	0.656	−0.080	0.153	−0.003	0.960	−0.040	0.458
Education level	−0.001	0.979	−0.006	0.906	0.019	0.740	−0.006	0.912
UKU I	**−0.175**	**0.012**	**−0.185**	**0.007**	−0.128	0.088	**−0.240**	**<0.001**
UKU II	0.025	0.666	−0.018	0.754	0.105	0.100	0.002	0.970
UKU III	−0.075	0.262	0.016	0.802	0.072	0.328	0.015	0.815
UKU IV	−0.023	0.732	−0.005	0.938	−0.059	0.410	0.051	0.421
MARS	**0.125**	**0.027**	**0.161**	**0.004**	**0.139**	**0.025**	**0.202**	**<0.001**
Diagnostic*	**0.154**	**0.013**	0.038	0.523	0.041	0.537	**0.116**	**0.047**
CGI-S	**−0.169**	**0.005**	**−0.269**	**<0.001**	**−0.186**	**0.004**	**−0.316**	**<0.001** ^a^

*1 Major Depressive Disorder, 2 Bipolar Disorder, 3 Schizophrenia.

^a^ *p* < 0.05 results in bold

Further multiple regression analyses were performed to control for treatments as confounders. The items ‘presence of antipsychotics’, ‘presence of SSRIs’ and ‘presence of benzodiazepines’ were added to the statistical model (see Table 4b in Supplementary Material).

Results were much the same. Age was a significant negative predictor of Relationships with Family (β = –0.232, *p* = 0.001) and Resilience (β = –0.174, *p* = 0.009). Psychic side effects (UKU I) were significant negative predictors across several quality of life domains, including Psychological Well-being (β = –0.255, *p* < 0.001), Resilience (β = –0.215, *p* = 0.002), Physical Well-being (β = –0.174, *p* = 0.013), Autonomy (β = –0.176, *p* = 0.010) and the overall SQoL-18 Index (β = –0.233, *p* < 0.001). Others Side Effects (UKU IV) were a significant positive predictor of Psychological Well-Being (β = 0.133, *p* = 0.038). Treatment adherence (MARS) positively predicted all the quality of life dimensions: Psychological Well-Being (β = 0.121, *p* = 0.027), Self-Esteem (β = 0.223, *p* < 0.001), Relationships with Family (β = 0.144, *p* = 0.017), Relationships with Friends (β = 0.132, *p* = 0.030), Resilience (β = 0.175, *p* = 0.002), Physical Well-Being (β = 0.128, *p* = 0.024), Autonomy (β = 0.162, *p* = 0.004), Sentimental Life (β = 0.135, *p* = 0.029) and the overall SQoL-18 Index (β = 0.203, *p* < 0.001). Diagnostic category also emerged as a significant predictor, with those diagnosed with MDD or bipolar disorder showing different patterns compared to those with schizophrenia. Illness severity (measured by the CGI-S) was a negative predictor of all the SQoL-18 dimensions (and the overall Index), except the Relationships with Family. Presence of SSRIs was only a negative predictor of Relationships with Friends (β = –0.116, *p* = 0.046) (Table 4b in Supplementary Material).

### Subgroup analyses by psychotropic medication classes

Subgroup analyses were conducted to identify which classes of psychotropic medications were associated with the most side effects.

Antipsychotics positively predict neurological side effects (UKU II), whether for typical (β = 0.140, *p* = 0.006), atypical (β = 0.139, *p* = 0.049), or both (β = 0.154, *p* = 0.029).

Antidepressants positively predict autonomic side effects (UKU III) (β = 0.144, *p* = 0.021), with a specificity of SNRIs on psychic side effects (UKU I) (β = 0.130, *p* = 0.042).

Benzodiazepines are significantly and positively associated with all the side effects explored (UKU I, II, III, IV) (β = 0.125 and *p* = 0.012 for UKU I, β = 0.161 and *p* = 0.002 for UKU II, β = 0.155 and *p* = 0.003 for UKU III and β = 0.196 and *p* ≤ 0.001 for UKU IV).

Others side effects (UKU IV) are positively predicted by typical antipsychotics (β = 0.166, *p* = 0.001) and mood stabilisers (β = 0.124, *p* = 0.015) (see Table 4a in Supplementary Materials).

## Discussion

This study provides critical insights into the perceived side effects of psychotropic medications, particularly those involving psychic symptoms like anxiety, depression and cognitive disturbances. A key challenge highlighted by our findings is the difficulty in distinguishing between these psychic side effects and symptoms inherent to the primary psychiatric disorder or comorbidities. For instance, patients often report anxiety and depressive symptoms as medication side effects (Correll 2007), which may actually be manifestations of their underlying condition. This issue is further complicated by the high prevalence of comorbid anxiety disorders in the sample.

Given this complexity, interpreting self-reported side effects requires careful consideration. Patients may mistakenly attribute their symptoms to medication rather than the disorder itself, potentially leading to inappropriate treatment adjustments or even non-adherence (Lambert et al. 2004). Addressing this challenge is crucial, and one way to mitigate these risks is through comprehensive psychoeducation. Educating patients about the potential side effects of their medication, while helping them differentiate these from symptoms of their illness, could improve treatment adherence and overall satisfaction.

### Key findings and study objectives

This study aimed to investigate the impact of psychotropic medication side effects on the quality of life in patients with severe mental illness (SMI), independent of treatment adherence, and to identify the drug classes most associated with side effects.

Our analysis showed that side effects, as identified by the UKU scores, were significant predictors of reduced quality of life across multiple domains, including multiple dimensions of QoL such as psychological well-being, resilience, physical well-being, autonomy and the overall SQoL-18 index. Importantly, these negative effects persisted independently of treatment adherence, as measured by the MARS scale. Antipsychotic medications were strongly linked to neurological side effects, while antidepressants, particularly SNRIs, were associated with increased psychic and autonomic side effects. Benzodiazepines, in particular, were associated with a wide range of side effects that detracted from quality of life, particularly in terms of autonomy and sentimental life.

Our results showed that ‘age’ was a significant negative predictor of ‘Relationship with Family’. We hypothesise that older age may reflect cumulative strain on familial relationships or differences in family support structures, which are well-documented in chronic mental illnesses. This finding underscores the need for targeted interventions to support family dynamics in older patients.

We also found that ‘other side effects’ (UKU IV) were a significant positive predictor of ‘Psychological Well-being’. This finding could be contradictive with another result: ‘psychic side effects’ (UKU I) were negative predictors across several domains of QoL. The side effects listed under ‘other side effects’ (UKU IV) are disparate and sometimes even contradictory (e.g. weight gain and weight loss). This lack of relationship between items should explain the contradictions in the results, while other UKU dimensions (I, II and III) seems to be more closely related.

Concerning relations between treatment adherence, MARS score positively predicted all the quality of life dimensions (Supplementary Materials, Table 4b) when controlling for psychotropic treatments (antipsychotics, SSRIs and benzodiazepines). This highlights that improved adherence is likely a proxy for enhanced patient engagement and symptom control, both of which contribute to better quality of life. This finding underscores the clinical importance of fostering adherence through psychoeducation and therapeutic alliance.

### Comparison with existing literature

The adverse impact of psychic and autonomic side effects on quality of life has been well-documented in prior studies (Pillinger et al. 2023). Our findings build on this body of research, particularly by demonstrating that these effects significantly impair multiple dimensions of quality of life, independent of adherence to treatment. Previous studies have shown that different antipsychotics have varying side effect profiles, with atypical antipsychotics frequently linked to metabolic side effects (De Hert et al. 2009; Siafis et al. 2018) like weight gain (Wu et al. 2022), diabetes (Holt 2019) and dyslipidemia (Weiden 2007). These findings extend to our results, showing that the adverse impact of side effects can persist across different psychotropic medication classes.

Our results regarding benzodiazepine use also align with literature emphasising the risks of dependence and withdrawal symptoms associated with long-term use (Lader 2014; Starcevic 2022). Cognitive impairments and reduced autonomy, both linked to benzodiazepine use in our study, have been highlighted in previous research (Lader 2014; Davies et al. 2017; Starcevic 2022), underscoring the need for cautious prescribing practices, particularly in polypharmacy settings (Citrome et al. 2005; Tiihonen et al. 2019; Lähteenvuo and Tiihonen 2021).

### Clinical implications

The findings of this study highlight the importance of managing psychotropic medication side effects to improve quality of life in patients with severe psychiatric disorders. Psychic side effects, often overshadowed by the focus on neurological and autonomic symptoms, emerged as a critical factor impacting quality of life. Clinicians should prioritise monitoring and mitigating these effects, which may involve selecting medications with more favourable side effect profiles or using adjunctive therapies to minimise their impact.

## Limitations and strengths

This study has several limitations. The cross-sectional design limits our ability to draw causal inferences between psychotropic side effects and quality of life. Longitudinal studies are needed to better establish the temporal relationship between these factors. Furthermore, our sample may not be fully representative of all patients with schizophrenia, depressive, or bipolar disorders, as we did not include other psychiatric conditions in our analysis. The reliance on self-reported measures also introduces the possibility of bias, though the use of validated instruments like the SQoL-18 and UKU scales helps mitigate this concern. Future studies could benefit from incorporating objective assessments of side effects, such as the Positive and Negative Syndrome Scale (PANSS) (Kay et al. 1987) or the Extrapyramidal Symptom Rating Scale (ESRS) (Chouinard 1980), to better differentiate between symptoms of the disorder and medication side effects. In addition, the confounding factors of psychiatric comorbidities have only been included in the statistical models presented in the Supplementary Tables (Table 4b), whereas these comorbidities deserve to be explored further.

Despite these limitations, the study’s large sample size provides robust statistical power and reliability. The comprehensive assessment of a wide range of side effects and quality of life domains offers a nuanced understanding of the challenges faced by patients with severe psychiatric disorders. Moreover, the inclusion of diverse diagnostic categories enhances the generalisability of the findings to clinical practice.

## Conclusion

In conclusion, this study emphasises the substantial burden that psychotropic medication side effects, particularly psychic symptoms, place on the quality of life of patients with severe psychiatric disorders. Our findings underscore the need for careful management of these effects to optimise treatment outcomes. Regular assessment of side effects and quality of life should be integrated into routine clinical practice to ensure that patients receive the most effective and least burdensome treatment possible. The importance of psychoeducation, individualised treatment plans and minimising polypharmacy where feasible cannot be overstated in improving both adherence and overall patient well-being. Concerning polypharmacy, the hypothesis is that as the number of different treatments increases, so do the UKU scores. This is consistent with the fact that a treatment with more different molecules probably indicates a worsening of the patient’s condition. And the more different molecules, the greater the risk of different side effects. According to our results, the negative impact on quality of life appears to be greater as side effects, measured by UKU scores, increase.

## Supplementary Material

supp mat review_bis.docx

Title_and_Authors_Korchia_et_al_130125 clean.docx
