# Supplementary material for: Impact of perceived side-effects of psychotropic treatments on quality of life in patients with severe mental illness
Source: Dialogues Clin Neurosci. 2025 Feb 11;27(1):10–9. doi: 10.1080/19585969.2025.2463443 (PMC11816623; doi:10.1080/19585969.2025.2463443)
Supplement: supp mat review_bis.docx [file TDCN_A_2463443_SM8798.docx]

Table 4b: multiple linear regressions analyses of relation between different side effects (UKU) and quality of life (SQoL-18)

| SQoL-18  Dimensions | Psychological  Well-Being | | Self Esteem | | Relations with  Family | | Relations with  Friends | | Resilience | |
| --- | --- | --- | --- | --- | --- | --- | --- | --- | --- | --- |
|  | standardized Bêta | (p) | standardized Bêta | (p) | standardized Bêta | (p) | standardized Bêta | (p) | standardized Bêta | (p) |
| Age | 0.021 | 0.738 | 0.047 | 0.458 | **-0.232** | **0.001** | 0.022 | 0.758 | **-0.174** | **0.009** |
| Sex | -0.020 | 0.713 | -0.039 | 0.483 | -0.046 | 0.451 | -0.036 | 0.563 | 0.082 | 0.159 |
| Education level | -0.008 | 0.874 | 0.019 | 0.707 | -0.042 | 0.465 | -0.045 | 0.442 | 0.021 | 0.708 |
| UKU I | **-0.255** | **<0.001** | -0.126 | 0.062 | -0.035 | 0.631 | -0.057 | 0.448 | **-0.215** | **0.002** |
| UKU II | -0.012 | 0.829 | -0.006 | 0.920 | 0.044 | 0.486 | -0.013 | 0.843 | -0.020 | 0.737 |
| UKU III | -0.025 | 0.695 | -0.017 | 0.797 | -0.024 | 0.735 | -0.034 | 0.642 | 0.022 | 0.749 |
| UKU IV | **0.133** | **0.038** | 0.048 | 0.462 | 0.075 | 0.292 | 0.001 | 0.990 | 0.064 | 0.344 |
| MARS | **0.121** | **0.027** | **0.223** | **<0.001** | **0.144** | **0.017** | **0.132** | **0.030** | **0.175** | **0.002** |
| Diagnostic* | **0.196** | **0.011** | **0.239** | **0.002** | **-0.233** | **0.005** | 0.022 | 0.794 | 0.040 | 0.607 |
| CGI-S | **-0.301** | **<0.001** | **-0.269** | **<0.001** | -0.084 | 0.186 | **-0.177** | **0.007** | **-0.189** | **0.002** |
| Antipsychotics | -0.001 | 0.987 | -0.005 | 0.945 | 0.127 | 0.112 | -0.113 | 0.157 | 0.017 | 0.826 |
| SSRIs | -0.081 | 0.131 | -0.091 | 0.093 | -0.108 | 0.068 | **-0.119** | **0.046** | -0.024 | 0.671 |
| Benzodiazepines | -0.005 | 0.929 | -0.039 | 0.470 | -0.056 | 0.354 | -0.006 | 0.919 | 0.055 | 0.333 |

| SQoL-18  Dimensions | Physical  Well-Being | | Autonomy | | Sentimental  Life | | SQoL-18 index | |
| --- | --- | --- | --- | --- | --- | --- | --- | --- |
|  | standardized Bêta | (p) | standardized Bêta | (p) | standardized Bêta | (p) | standardized Bêta | (p) |
| Age | -0.113 | 0.086 | -0.106 | 0.097 | 0.042 | 0.547 | -0.088 | 0.153 |
| Sex | 0.025 | 0.667 | -0.085 | 0.131 | 0.001 | 0.993 | -0.043 | 0.422 |
| Education level | <0.001 | 0.994 | -0.013 | 0.804 | 0.035 | 0.552 | -0.002 | 0.976 |
| UKU I | **-0.174** | **0.013** | **-0.176** | **0.010** | -0.129 | 0.088 | **-0.233** | **<0.001** |
| UKU II | 0.032 | 0.594 | -0.010 | 0.866 | 0.083 | 0.196 | 0.005 | 0.936 |
| UKU III | -0.077 | 0.250 | 0.019 | 0.777 | 0.073 | 0.317 | 0.014 | 0.826 |
| UKU IV | -0.021 | 0.751 | 0.007 | 0.916 | -0.062 | 0.395 | 0.057 | 0.370 |
| MARS | **0.128** | **0.024** | **0.162** | **0.004** | **0.135** | **0.029** | **0.203** | **<0.001** |
| Diagnostic* | **0.160** | **0.041** | 0.012 | 0.879 | -0.046 | 0.587 | 0.075 | 0.309 |
| CGI-S | **-0.168** | **0.005** | **-0.262** | **<0.001** | **-0.199** | **0.002** | **-0.320** | **<0.001** |
| Antipsychotics | -0.041 | 0.588 | 0.017 | 0.818 | 0.146 | 0.078 | 0.031 | 0.666 |
| SSRIs | -0.067 | 0.230 | -0.057 | 0.297 | 0.018 | 0.763 | -0.084 | 0.111 |
| Benzodiazepines | 0.006 | 0.913 | -0.075 | 0.177 | 0.046 | 0.450 | -0.008 | 0.886 |

(*) 1 Major Depressive Disorder, 2 Bipolar Disorder, 3 Schizophrenia
