# Supplementary material for: Impact of perceived side-effects of psychotropic treatments on quality of life in patients with severe mental illness
Source: Dialogues Clin Neurosci. 2025 Feb 11;27(1):10–9. doi: 10.1080/19585969.2025.2463443 (PMC11816623; doi:10.1080/19585969.2025.2463443)
Supplement: Title_and_Authors_Korchia_et_al_130125 clean.docx [file TDCN_A_2463443_SM8797.docx]

**Impact of perceived side-effects of psychotropic treatments on quality of life in patients with severe mental illness**

Théo Korchia ^1, 2^ *, Mélanie Faugère ^1, 2^, Vincent Achour ^1, 2^, Eloïse Maakaron ^1, 2^, Christelle Andrieu-Haller ^1, 2^, Guillaume Fond ^1, 2^, Christophe Lançon ^1, 2^

^1^ Department of University Psychiatry, Sainte Marguerite University Hospital, Assistance Publique des Hôpitaux de Marseille, Marseille, France

^2^ Assistance Publique des Hôpitaux de Marseille, Aix-Marseille University, UR3279: Health Service Research and Quality of Life Center – CEReSS, Marseille, France

* Address of the corresponding author: Service du Pr Christophe Lançon, CHU Sainte Marguerite, Pavillon Solaris, 270 boulevard de Sainte Marguerite, 13009, Marseille, France.

E-mail address: [theo.korchia@ap-hm.fr](mailto:theo.korchia@ap-hm.fr) *(*T. Korchia)
